# Inattention over time-on-task: the role of motivation in mitigating temporal increases in media multitasking

**DOI:** 10.3389/fcogn.2025.1547295

**Published:** 2025-05-06

**Authors:** Allison C. Drody, Effie J. Pereira, James Danckert, Daniel Smilek

**Affiliations:** ^1^Department of Psychology, University of Waterloo, Waterloo, ON, Canada; ^2^Department of Psychology, Queens University, Kingston, ON, Canada

**Keywords:** media multitasking, motivation, attention, vigilance, time-on-task

## Abstract

**Introduction:**

Numerous studies have demonstrated that attention and performance decline with time-on-task. In modern contexts, this gradual fading of attention can manifest as increases in media multitasking over time. Across two studies, we investigated whether increasing individuals' motivation to perform well on a task mitigates temporal increases in media multitasking.

**Method:**

Study 1 re-analyzed data from a previously published study which provided participants with standard or motivating instructions before having them complete a sustained attention task with the option to media multitask. Study 2 extended this work by critically assessing in-the-moment motivation through thought probes throughout the task.

**Results:**

In both studies, media multitasking and corresponding decreases in task performance over time were attenuated as a function of increased motivation. Moreover, results from Study 2 revealed that motivation decreased with time-on-task for both groups; however, this decline was more gradual in the motivated group.

**Discussion:**

Our findings suggest that increasing the value individuals assign to attending to their current task may aid in prolonging sustained attention. These findings align with recent theories of vigilance that attribute temporal decrements in attention and performance to varying cost-benefit analyses rather than a depletion of resources over time.

## 1 Introduction

It is a universal human experience that when completing a task for extended periods of time, one's attention begins to fade along with one's ability to perform well on the task. This experience was first formally studied by Mackworth (1948), who sought to understand why radar operators in the Second World War became less efficient as their shifts progressed. Employing a task that mimicked the demands of radar surveillance by requiring participants to monitor a screen for extended periods of time while responding to infrequent targets, Mackworth (1948) found that individuals became more likely to miss targets as time-on-task increased. Numerous studies have since observed similar reductions in performance over time (Grier et al., 2003; Helton and Warm, 2008; Pattyn et al., 2008; Helton and Russell, 2011), which are often accompanied by self-reported decreases in attention (Risko et al., 2012; Thomson et al., 2014; Yanko and Spalek, 2014; Krimsky et al., 2017; Brosowsky et al., 2023). This phenomenon, known as the vigilance or sustained attention decrement, has commonly been understood from a resource-depletion perspective, which suggests that the cognitive resources required to effectively attend to a task diminish with time-on-task, leading to gradual declines in performance (Smit et al., 2004; Warm et al., 2008; Helton and Russell, 2011; Greenlee et al., 2024).

In more modern contexts, where digital media devices are ubiquitous, sustained attention is commonly interrupted by the temptation to engage with these devices (Rosen et al., 2013; Brasel and Gips, 2017; Demirbilek and Talan, 2018). Media multitasking, the simultaneous and typically non-required completion of multiple tasks when at least one of the tasks involves media (e.g., watching Netflix while writing a manuscript; Wang and Tchernev, 2012), may therefore provide a valuable lens through which to examine sustained attention decrements. Recent studies have explored how media multitasking changes over the course of a primary task by allowing participants to voluntarily engage in media activities during course lectures (Ragan et al., 2014; Wammes et al., 2019) and cognitive laboratory tasks (Drody et al., 2024; Ralph et al., 2020, 2021), or to switch between and within multiple forms of media at their discretion (e.g., videos, online articles; Tam and Inzlicht, 2024). Some of these studies have found that media multitasking increases with time-on-task and that these increases may be detrimental to task performance. For instance, Wammes et al. (2019) asked students to self-report whether they were media multitasking at various points during a class lecture, finding small but significant increases in the proportion of times students reported media multitasking over the course of the lecture. They additionally found that higher rates of media multitasking were negatively associated with lower scores on a quiz based on the lecture content. In the laboratory, Drody et al. (2024) tracked whether participants media multitasked by engaging with a task-irrelevant video during each trial of a sustained attention task. The authors noted that the proportion of participants who media multitasked with the video rose from ~18% at the start of the task to 37% by the end. This increase in media multitasking was accompanied by decreases in task performance (e.g., a 9–16% reduction in hit rate, depending on condition). Intriguingly, these findings closely resemble the reductions in attention and task performance observed in prior sustained attention research (e.g., Brosowsky et al., 2023; Davies and Parasuraman, 1982; Helton and Russell, 2011; Krimsky et al., 2017; Mackworth, 1948; Risko et al., 2012; Smit et al., 2004; Thomson et al., 2014; Yanko and Spalek, 2014).

A small number of studies have also noted non-linear fluctuations in media multitasking over time. For instance, Rosen et al. (2013) observed participants' behaviors while they studied for a period of 15-min and found that the percentage of time they spent engaged in off- vs. on-task activities varied throughout the session. Media multitasking was highest near the start and middle of the session and decreased steeply near the end. Additionally, Ragan et al. (2014) monitored students' laptop usage during a 3-h lecture and observed that off-task laptop use peaked near the start and middle of the lecture before declining toward the end. Although these fluctuations do not closely resemble standard sustained attention decrements, they may offer important theoretical insights when considered alongside other research on temporal changes in media multitasking. If declines in attention and performance over time are caused by a gradual depletion of resources, one might expect individuals to become less inclined to engage in additional activities (e.g., media multitasking) as their task progresses; however, findings from recent media multitasking research do not follow this pattern. Instead, they align with the perspective that the degree to which an individual attends to a primary task likely depends on a variety of transient factors, such as their current levels of motivation (Ralph et al., 2021) or interest in the task-at-hand (Deng et al., 2024), rather than a mere depletion of resources over time.

Compatible with this view is Kurzban et al.'s (2013) opportunity costs account of mental effort. This account holds that, because individuals are capable of engaging in a limited number of tasks at any given time, they must prioritize between the numerous tasks available to them by continually monitoring opportunity costs. That is, the value of completing a current task compared to the cost of being unable to engage in others. Kurzban et al. (2013) explain that the value that can be gained from persisting with a single task generally decreases over time, while value that could be gained from engaging with alternatives increases. Thus, opportunity costs are theorized to increase with time-on-task. The authors further posit that states such as boredom signal rising opportunity costs, leading individuals to shift their attention elsewhere (see also Struk et al., 2020). From this perspective, prolonged task completion should be associated with rising feelings of boredom and an increasing likelihood of engaging in other activities. Because attention to the task should diminish over time, decreases in effort devoted to the primary task and declines in task performance would also be expected.

Drody et al. (2024) applied this framework to media multitasking, hypothesizing that increases in boredom over the course of a primary task, reflecting naturally rising opportunity costs, could lead to media multitasking when individuals have access to digital media. In their study, participants completed a sustained attention task while having the option to media multitask by simultaneously playing a video lecture that they could turn on or off at any time during the experiment. This design allowed the researchers to use instances when the video lecture was turned on to track changes in media multitasking over time. The authors also probed participants' levels of boredom and the amount of effort they devoted to the sustained attention task throughout the experimental session. In line with the opportunity costs account, Drody et al. (2024) found that boredom increased with time-on-task (with increases ranging from ~30 to 38% depending on condition), and that this increase was accompanied by a 19% increase in media multitasking. Moreover, the amount of effort participants devoted to the task decreased over time along with task performance (11–23% and 9–16% decreases, respectively, depending on condition).

Further evidence for a link between boredom and media multitasking has been found in a series of recent experiments by Tam and Inzlicht (2024). In one of these experiments, participants experienced two phases of video watching: a switching phase, during which they were presented with several videos which they could switch between at their discretion, and a no-switching phase, during which they watched a single video. The order of these phases differed between conditions. Specifically, in the low opportunity costs condition, participants experienced the no-switching phase followed by the switching phase. In contrast, in the high opportunity costs condition, participants experienced the switching phase followed by the no-switching phase. The rationale was that the perceived opportunity costs of the no-switching phase would be particularly high for participants in the high opportunity cost condition, as experiencing the switching phase first would likely make them aware that they were missing out on the opportunity to engage with other content. Consistent with this notion, during the no-switching phase, participants in the high opportunity costs condition reported moderately higher levels of boredom than participants in the low opportunity costs condition. Moreover, participants in the high opportunity costs condition reported moderately greater perceived opportunity costs during the no-switching phase compared to the switching phase, indicating that they felt they were missing out on other videos they would have liked to watch. Taken together, these findings provide further evidence for the notion that temporal changes in attention are driven more by momentary changes in variables tied to opportunity costs, such as boredom, than by a depletion of cognitive resources.

From this perspective, it should be possible to mitigate temporal increases in media multitasking by manipulating variables relevant to one's opportunity cost calculations. Given that media multitasking is a highly prevalent behavior (e.g., Junco and Cotten, 2012; Voorveld and van der Goot, 2013) associated with reduced performance across various domains, including reading (Bowman et al., 2010; Jeong and Hwang, 2012; Lee et al., 2012), studying (Patterson, 2017), attending to lectures (Demirbilek and Talan, 2018; Wammes et al., 2019; Jamet et al., 2020), and completing cognitive tasks in the laboratory (Drody et al., 2022, 2024; Lopez and Orr, 2022; Ralph et al., 2020, 2021), many studies have explored possible interventions for curbing this behavior. These interventions have included educating individuals about the negative consequences of multitasking (Tassone et al., 2020), increasing individuals' awareness of their media use habits (Adler et al., 2015; Whittaker et al., 2016), or blocking access to distracting media (Kim et al., 2019a,b; see Biedermann et al., 2021 for a review of media multitasking interventions); however, many of these interventions have shown limited success. One possible explanation for this limited success is that they failed to sufficiently influence variables related to individuals' opportunity cost calculations. For example, while educational interventions may increase individuals' understanding of the negative consequences of media multitasking, they may not affect the value they assign to attending to a primary task (e.g., the importance of performing well on the task), which may remain low, vs. the benefits they associate with media multitasking (e.g., entertainment; Hwang et al., 2014; Kononova and Chiang, 2015; Kononova and Yuan, 2017), which may remain high.

Altering individuals' levels of motivation, which should directly affect the value they assign to attending to their current task vs. alternative tasks, may be more effective in this regard. In line with this view, individuals may be more willing to exert effort to achieve highly valued outcomes and may experience this effort as less aversive than when the outcome is not valued (Inzlicht et al., 2018). Prior work has supported this notion by illustrating that higher levels of motivation are associated with greater attention to one's primary task (Engelmann and Pessoa, 2007; Robinson et al., 2012; Seli et al., 2015, 2019; Walsh et al., 2021), and decreases in motivation with time-on-task are shown to coincide with reductions in attention (Brosowsky et al., 2023). To investigate how increasing motivation influences media multitasking, Ralph et al. (2021) employed a similar paradigm to that of Drody et al. (2024) by having participants complete a sustained attention task with the option to media multitask by simultaneously playing a video lecture. Critically, Ralph et al. (2021) had participants complete the study in one of two conditions: one in which they received standard task instructions and another in which they were motivated by being told that they could leave the experimental session early if they performed sufficiently well on the task. The authors found that participants who received the motivating instructions media multitasked significantly less than those given standard instructions. Whereas, the median number of media multitasking trials in the group who received standard instructions was ~212, the median number of media multitasking trials in the motivated group was 15. These results provide support for the notion that increasing motivation reduces rates of media multitasking. However, no studies to our knowledge have examined how the effect of increasing motivation unfolds over time.

Momentary shifts in motivation could influence media multitasking over time, and perhaps more broadly attention over time, in a variety of possible ways. For example, given that media multitasking increases with time-on-task (Wammes et al., 2019; Drody et al., 2024), increasing motivation might act to reduce this behavior for the entire duration of a task. A strong account of the effects of motivation would suggest that this would eliminate the vigilance decrement. A more moderate hypothesis would suggest that motivation attenuates the vigilance decrement making any decrease in performance less dramatic when compared with conditions in which motivation is not increased. Finally, it is also possible that manipulating motivation leads to only short-lived effects on media multitasking, consistent with prior work demonstrating that the effects of mood inductions often do not persist for longer than ~5 min (Kuijsters et al., 2016; Hunter and Eastwood, 2018; Gillies and Dozois, 2021; Drody et al., 2022; Monno et al., 2024). This could lead to differences in aggregate levels of media multitasking between groups but may call into question the utility of such interventions. These different outcomes highlight the importance of examining how increasing motivation influences changes in media multitasking over time.

Across two studies, we investigated how increasing individuals' motivation to complete a primary task influences temporal changes in media multitasking. In the first study, we reanalyzed data from Ralph et al.'s (2021) experiment in which participants received standard or motivating task instructions before completing a sustained attention task with the option to media multitask. Our reanalysis aimed to elucidate how increasing motivation influenced participants' media multitasking behaviors over the course of the experiment. In the second study, we extended this work by incorporating thought probes to assess motivation throughout the primary task. This allowed us to better evaluate how motivation and media multitasking changed as the task progressed. We anticipated that media multitasking would increase with time-on-task and that motivation and task performance would decrease. Additionally, we expected to observe differences between the groups who received standard vs. motivating task instructions. Specifically, we predicted that participants who received the motivating instructions would begin the task with higher levels of motivation and experience more gradual declines in motivation over time compared to those who received the standard instructions. We further anticipated that participants who received the motivating instructions would show more gradual increases in media multitasking as well as more gradual decreases in task performance over time compared to those who received the standard instructions.

## 2 Study 1

In our first study, we re-analyzed data from Ralph et al.'s (2021) Experiment 1 to investigate how increasing participants' motivation influenced their media multitasking behaviors over the course of the task. We begin with a brief overview of Ralph et al.'s (2021) methods and data pre-processing procedures, followed by a summary of their original analyses relevant to our research question. Finally, we examine how media multitasking behaviors changed over time depending on whether participants received motivating or standard task instructions.

### 2.1 Materials and methods

#### 2.1.1 Participants

Ralph et al. (2021) collected a total of 166 participants, aged 17–35 years old (*M*_*age*_ = 19.43; *SD*_*age*_ = 2.47), from a SONA undergraduate participant pool at the University of Waterloo between the Fall 2017 and Winter 2018 semesters. The sample size was determined via an a priori stopping rule for data collection, whereby they aimed to recruit a total of 80 participants per condition. Participants were tested in the laboratory in varying groups of 1–5 and received course credit in exchange for their participation. Prior to data pre-processing, the Control condition included 84 participants, while the Motivated condition included 82 participants.^1^

#### 2.1.2 1-back task and media multitasking

The 1-back in Ralph et al. (2021) consisted of 486 trials and lasted ~20-min. Eighteen of these trials were practice trials that were removed prior to data analysis, and the remaining 468 trials were experimental trials. On each trial, a single letter (B, F, K, H, M, Q, R, X, Z) was randomly selected and displayed in the center of the screen for 500 ms, followed by a 2,000 ms fixation cross. The next stimulus appeared immediately after the presentation of the fixation cross and marked the start of a new trial. Participants were asked to respond via a keypress when the letter on the screen matched the letter presented on the previous trial and to withhold a response when the letters did not match. Responses were recorded for a given trial if they were provided any time during the presentation of the stimulus or the subsequent fixation cross. Participants were not provided with feedback following their responses as drawing additional attention to their performance in this manner could have biased their willingness to media multitask. This task was completed continuously, without breaks except brief pauses to respond to thought probes (see below). Performance was assessed in terms of proportion hits (i.e., the proportion of correct responses to target trials) and false alarms (i.e., the proportion of incorrect responses to non-target trials).

**Figure 1:**
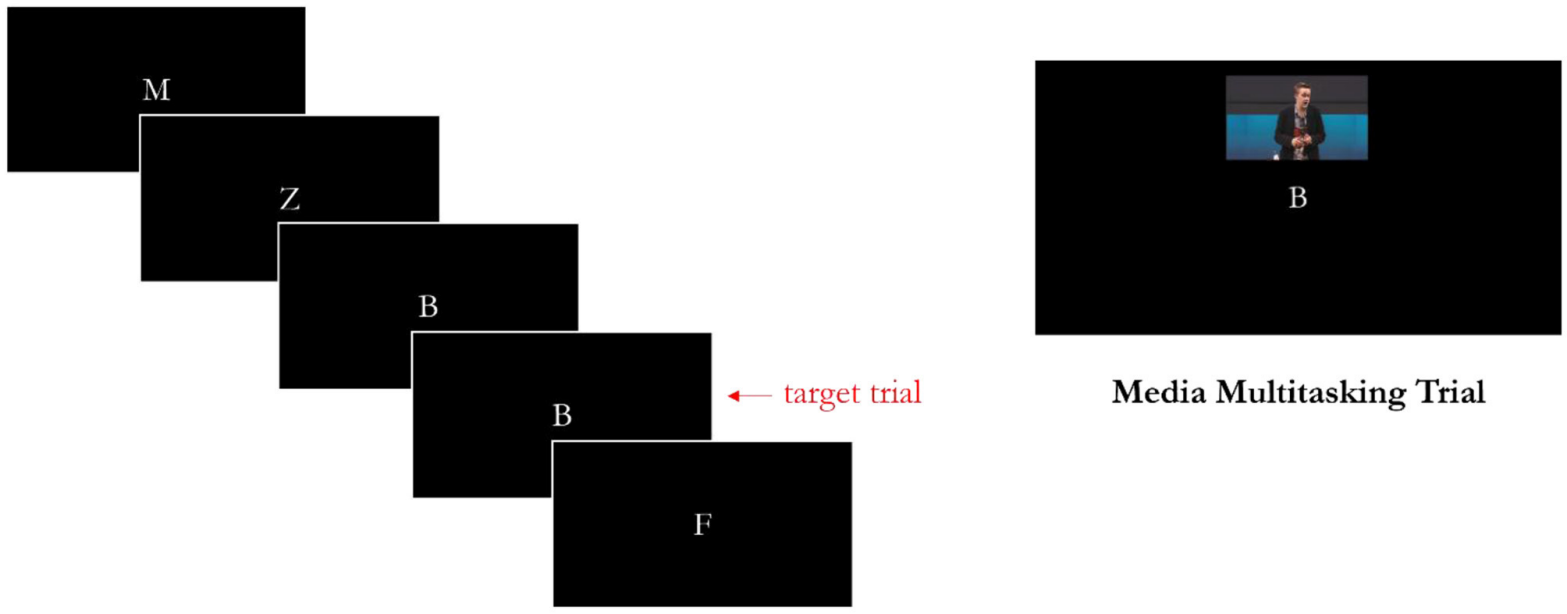
Depiction of a sequence of 1-back trials with the video off **(left)** and an example of a single media multitasking trial with the video on **(right)**.

While completing the 1-back, participants were given the opportunity to media multitask by playing a task-irrelevant video (a TED talk called Brain Magic; Barry, 2004). The video could be turned on or off at any point throughout the task by pressing the “t” key. When played, the video appeared in the center of the screen above the 1-back stimuli (Figure 1). Media multitasking was calculated by summing the number of trials participants spent with the video on.

#### 2.1.3 Motivation manipulation

Before completing the 1-back task, participants in Ralph et al.'s (2021) study were randomly assigned to either a Control or Motivated condition. In the Control condition, participants received standard 1-back instructions, while in the Motivated condition, they received the following instructions informing them that they could leave the experimental session early if they performed sufficiently well on the task:

“Before you begin, there is one more thing you should know.

That is, as you know, this is a one-hour long study. However, depending on how well you do on the task, you may be able to leave about halfway through the task while still earning the full credit.

To determine whether you get to leave early, during the task, the computer will monitor your performance on the task. After about 20 min, the task will temporarily stop, and the computer will compute your overall performance on the task up until that point, and then you will be notified if you have achieved a high enough level of performance to be let out of the study early while still receiving the full participation credit. If you do not achieve a high enough level of performance on the task, then you will have to complete the task for an additional 20 min, for a total time of nearly 1 h, as initially stated on SONA.

One thing that is very important to note is that your decision to watch the video WILL NOT be considered when the computer analyzes your performance on the task.

Do you have any questions?”

In reality, the study length was identical across conditions, such that participants in both groups only completed 20 min of the 1-back task.

#### 2.1.4 Self-reported motivation questions

A question previously employed by Seli et al. (2019) was used to probe motivation immediately after participants received the standard or motivating task instructions as well as after they completed the 1-back task. Participants were asked, “How motivated [are/were] you to do well on the primary task?” and responded on a Likert scale with response options ranging from 1 (not motivated at all) to 7 (very motivated).

#### 2.1.5 Familiarity with the video

Participants' familiarity with the video was assessed using the question, “Have you seen the video presented in this study before?”. Participants could respond either “Yes, I have seen this video before”, “No, I have not seen this video before”, or “N/A, I did not watch the video”. If a large proportion of participants had previously seen the video, this might have influenced their media multitasking patterns; however, only one participant responded “Yes” to this question. Therefore, responses to this question were not considered when analyzing the data.

### 2.2 Results

#### 2.2.1 Data pre-processing

Data pre-processing procedures remained the same as those reported by Ralph et al. (2021). That is, prior to data analysis, participants with < 30% hits or >20% false alarms were removed from the dataset, as exceptionally poor performance on the 1-back raises concerns about whether participants understood the task instructions or were simply unwilling to comply with them. After removing nine participants who met these criteria, the sample consisted of 157 participants (44 male, 112 female, 1 non-binary) with an age range of 17 to 35 (*M*_*age*_ = 19.50, *SD*_*age*_ = 2.51). There were 78 participants in the Control condition and 79 in the Motivated condition.

#### 2.2.2 Impact of manipulation on motivation

**Figure 2:**
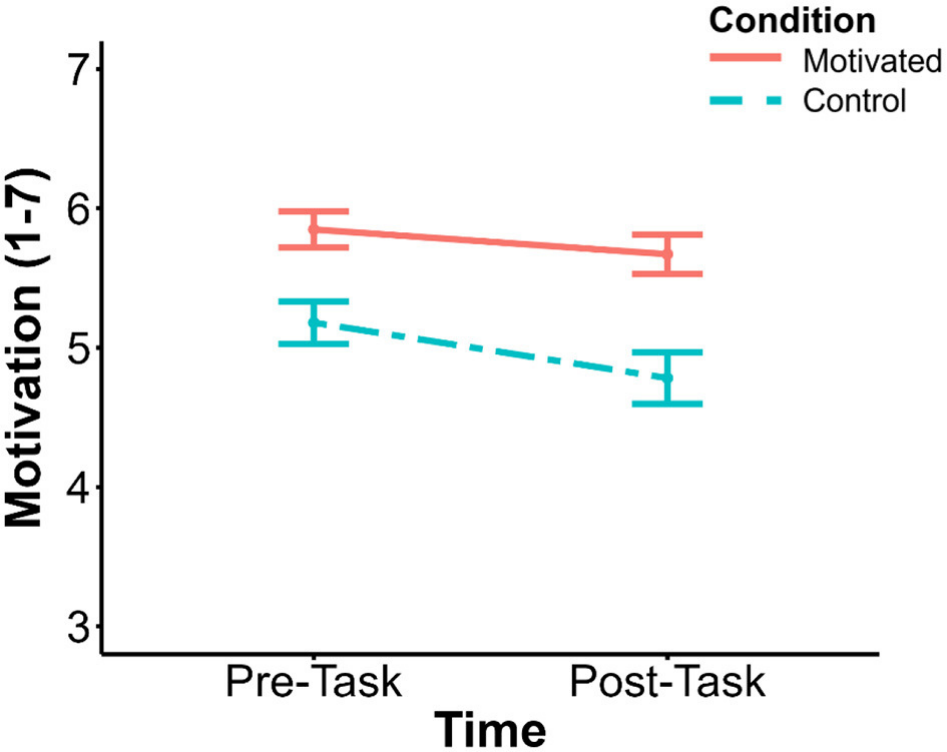
Graph depicting mean levels of motivation before and after participants completed the 1-back. Error bars represent +/– 1 standard error of the mean.

Changes in motivation from the start to the end of the task are illustrated in Figure 2. Results of a mixed factorial analysis of variance (ANOVA) with Condition (Motivated vs. Control) as a between-participants factor and Time (Pre-Task vs. Post-Task) as a within-participants factor indicated that there was a main effect of Condition, *F*_(1,155)_ = 15.86, *p* < 0.001, $\eta_{p}^{2}$ = 0.09, with participants in the Motivated condition reporting higher levels of motivation than participants in the Control condition. There was also a main effect of Time *F*_(1,155)_ = 9.21, *p* = 0.003, $\eta_{p}^{2}$ = 0.06, such that participants were more motivated at the start of the task compared to the end of the task. There was no significant interaction between Condition and Time, *F*_(1,155)_ = 1.35, *p* = 0.25, $\eta_{p}^{2}$ = 0.01.

#### 2.2.3 Impact of motivation on media multitasking

**Figure 3:**
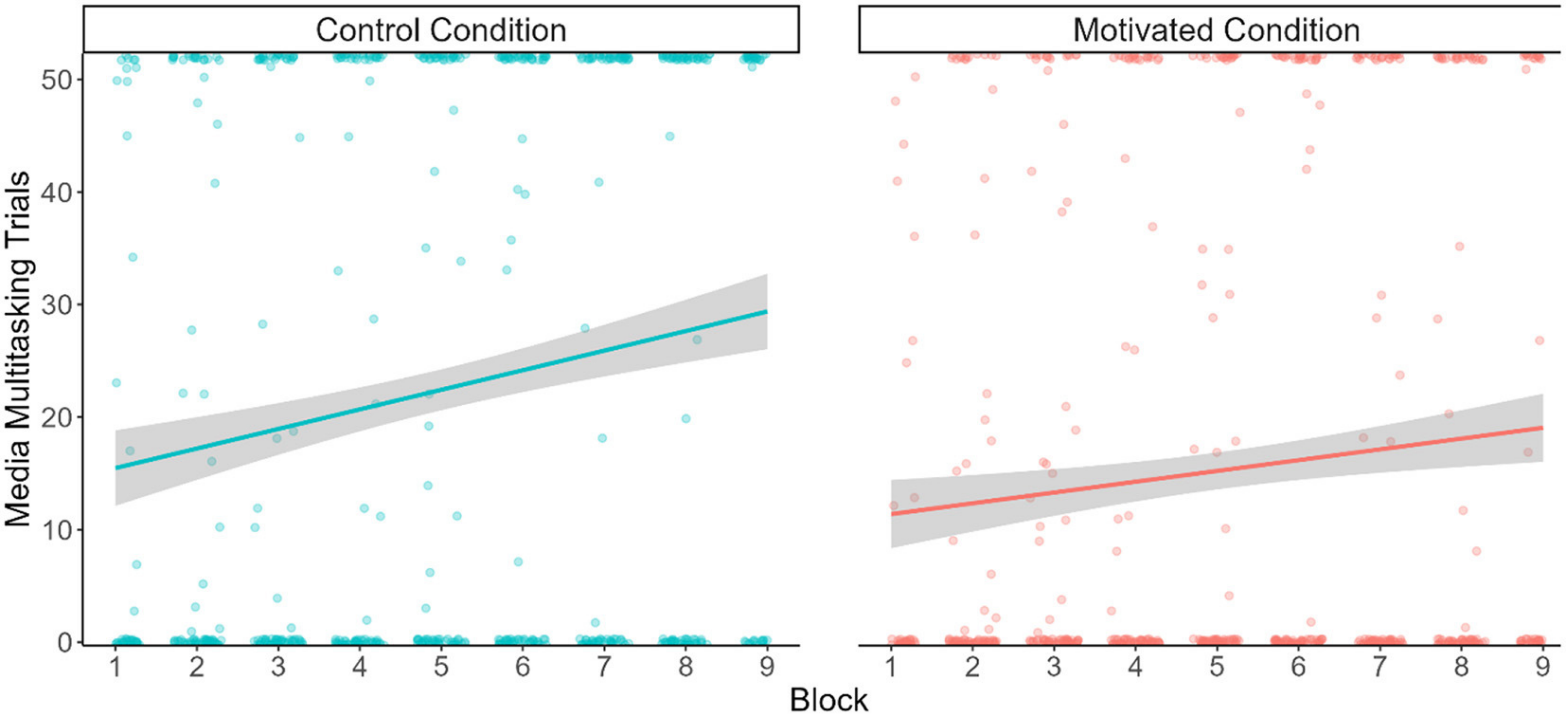
Scatterplots depicting changes in media multitasking over successive blocks of the 1-back for participants in the Control condition **(left)** and Motivation condition **(right)**. Data points are jittered to increase their visibility. Regression lines are fitted to the data and shading represents 95% confidence intervals.

To examine changes in media multitasking over the course of the 1-back, we divided the task into nine blocks of 52 trials and calculated the sum of the trials participants spent with the video on for each block. A Generalized Linear Mixed Model (GLMM) was then employed to determine whether patterns of media multitasking throughout the 1-back varied across blocks and motivation conditions. This approach was chosen for two key reasons. First, the media multitasking data were highly negatively skewed (Figure 3). While the majority of participants played the video at some point during the study, 36% of participants in the Control condition never played the video whereas a higher percentage (46%) of participants in the Motivated condition chose not to play the video. Second, we anticipated that participants' likelihood of playing the video in a given block would be influenced by whether they had done so in previous blocks. That is, levels of media multitasking were likely to be correlated across blocks.

The GLMM allowed us to address these issues by specifying a non-normal distribution for our outcome variable and allowing changes in media multitasking over the nine blocks to vary across participants. In our model, Condition (with the control condition as the reference group) and Block (centered around zero) were included as fixed effects. The intercept and slope for Block were allowed to vary across participants, and a Poisson distribution was specified for our outcome variable (i.e., number of trials with media multitasking).

As shown in Table 1, results of the GLMM revealed that, while there was no effect of Condition or Block on media multitasking, there was a significant interaction between these factors, indicating that participants in the Motivated condition showed more gradual increases in media multitasking over time compared to participants in the Control condition (Figure 3). More information about participants' media multitasking behaviors can be found in the Supplementary material.

**Table 1:** Generalized linear mixed model predicting changes in media multitasking based on block and condition.

**Fixed effects**	**Estimate**	**SE**	***z***	***p***
Intercept	−0.45	0.62	−0.72	0.469
Condition (motivated group)	−1.39	0.85	−1.63	0.104
Block	−0.09	0.08	−1.20	0.231
Block^*^Condition	−0.18	0.08	−2.17	0.030

N = 157. Coefficients for the effect of Condition are shown for the Motivated condition (Control is the reference group). P-values are based on Wald z-tests comparing estimates against zero.

#### 2.2.4 Changes in task performance over time

**Figure 4:**
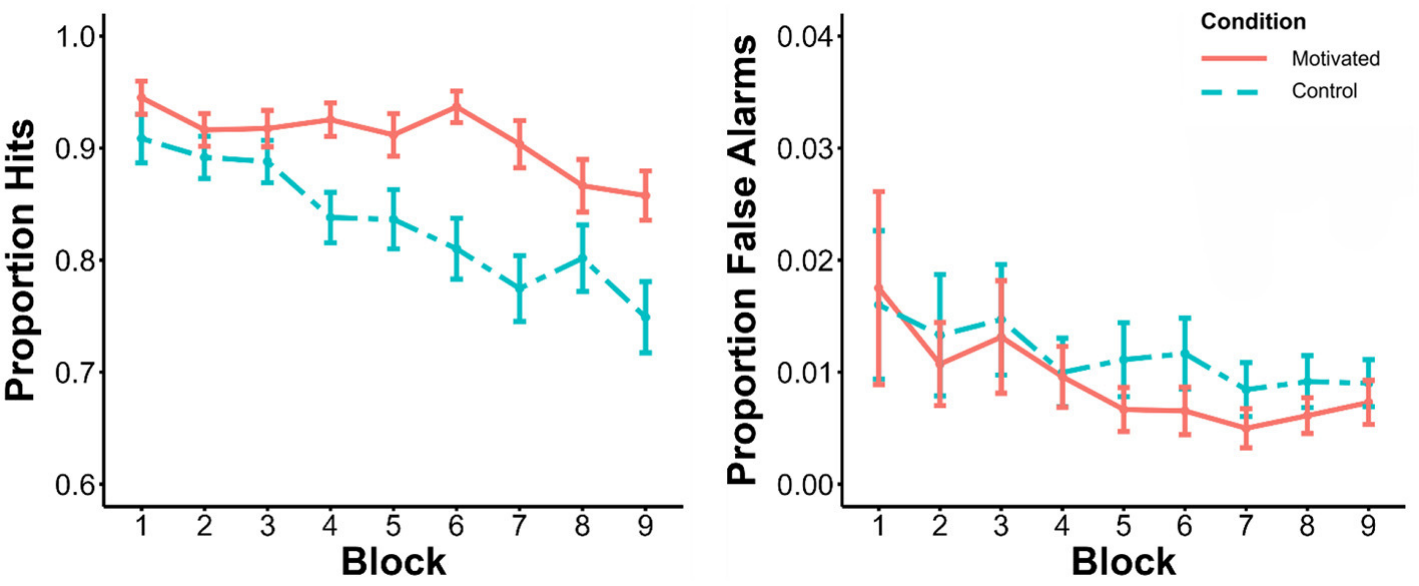
Line graphs depicting proportion hits **(left)** and false alarms **(right)** over successive blocks of the 1-back as a function of condition. Error bars represent +/– 1 standard error of the mean.

To assess how performance changed over the course of the 1-back, proportion hits and false alarms were computed for each of the nine blocks of 52 trials in the 1-back task. These data are shown in Figure 4. Two mixed factorial ANOVAs were then conducted with Condition as a between-participants factor, Block as a within-participants factor, and either proportion hits or proportion false alarms as the dependent variable. Mauchly's tests indicated that the assumption of sphericity was violated for both proportion hits (*W* = 0.27, *p* < 0.001) and proportion false alarms (*W* = 0.00, *p* < 0.001), therefore results are presented after applying a Greenhouse-Geisser correction (ε _*proportion hits*_ = 0.69, ε _*proportion false alarms*_ = 0.28).

For proportion hits, there was a main effect of Condition, *F*_(1,155)_ = 10.81, *p* < 0.001, $\eta_{p}^{2}$ = 0.07, with participants in the Control condition displaying lower proportion hits than participants in the Motivated condition. There was also a main effect of Block, *F*_(5.55,860.04)_ = 12.23, *p* < 0.001, $\eta_{p}^{2}$ = 0.07, indicating that proportion hits declined over time-on-task. Additionally, there was a significant interaction between Condition and Block, *F*_(5.55,860.04)_ = 3.24, *p* = 0.005, $\eta_{p}^{2}$ = 0.02, such that participants in the Motivated condition displayed more gradual declines in performance over time than participants in the Control condition.

For proportion false alarms, there was no main effect of Condition, *F*_(1,155)_ = 0.39, *p* = 0.535, $\eta_{p}^{2}$ < 0.01, or Block, *F*_(2.26,349.84)_ = 2.18, *p* = 0.108, $\eta_{p}^{2}$ = 0.01, nor was there a significant interaction between Condition and Block, *F*_(2.26,349.84)_ = 0.21, *p* = 0.834, $\eta_{p}^{2}$ < 0.01. Analyses examining the effect of media multitasking on task performance can be found in the Supplemental material.

### 2.3 Discussion

Our first study re-analyzed data from Ralph et al. (2021) to investigate how increasing motivation influences patterns of media multitasking over time. In line with our hypotheses, we found that temporal increases in media multitasking were attenuated for participants in the Motivated condition compared to those in the Control condition. Moreover, consistent with the notion that media multitasking negatively impacts task performance, decreases in performance over time were more gradual in the Motivated condition relative to the Control condition. These findings align with the view that increasing participants' motivation to complete a task can help them delay engaging in other activities like media multitasking for longer than they otherwise would.

## 3 Study 2

In Study 2, we aimed to replicate and extend the findings from Study 1 by critically assessing in-the-moment changes in motivation throughout the 1-back task in order to understand how manipulating motivation would impact media multitasking, performance, and self-reported measures of motivation over time-on-task. We predicted that patterns of media multitasking and performance would remain similar to those observed in Study 1, with participants in the Motivated condition displaying more gradual increases in media multitasking and slower declines in performance over time compared to those in the Control condition. We further anticipated that self-reported levels of motivation would decline over the course of the 1-back task and that these changes would be more gradual in the Motivated condition than in the Control condition.

In addition to in-the-moment assessments of motivation throughout the task, for exploratory purposes, we also included additional post-task questions assessing participants' experiences (i.e., interest and enjoyment) of the 1-back. We specifically chose to measure interest and enjoyment because these experiences may play a role in one's intrinsic motivation to complete a task. To gain a deeper understanding of the effect of our motivation manipulation, we also asked participants in the Motivated group how motivated they were to complete the task given our motivating instructions.

### 3.1 Materials and methods

#### 3.1.1 Participants

We aimed to collect a larger sample in Study 2 for several reasons. First, we wanted to account for the possibility that rates of media multitasking would be low. Media multitasking occurred infrequently in Study 1, which was conducted in-person, and prior work has shown that participants commonly engage in off-task activities during online studies (Drody et al., 2023). This raised the possibility that the online design of Study 2 might encourage off-task activities with content unrelated to the experiment, further reducing media multitasking with the video lecture. Thus, it was important that we maximize our chances of capturing enough instances of media multitasking to support our analyses. Second, this study was designed to reveal possible temporal effects, which required the use of a GLMM for analysis. These models parse variance across a greater number of parameters than more traditional statistical models (e.g., ANOVAs), necessitating a large sample size. To ensure that we could collect a sufficiently large sample, we implemented a stopping rule that constrained data collection to the duration of the Fall 2022 semester.

A total of 428 participants (1 agender, 6 genderqueer, 76 men, 339 women, 6 no answer provided) were recruited from a SONA online participant pool at the University of Waterloo. Two hundred and eighteen participants were assigned to the Motivated condition and 210 participants were assigned to the Control condition. Participants were provided a link to the study website and completed the study online in exchange for course credit. Based on responses from the 414 participants who provided information regarding their age, our sample ranged between 17 and 44 years of age (*M*_*age*_ = 20.04; *SD*_*age*_ = 3.36).

#### 3.1.2 1-back task and media multitasking

The media multitasking paradigm was identical to that of Ralph et al. (2021). That is, participants completed a 1-back task during which they could turn a video (the TED talk on Brain Magic; Barry, 2004) on or off at their discretion. Media multitasking was once again operationalized as the number of trials participants spent with the video on.

#### 3.1.3 Motivation manipulation

As in Ralph et al. (2021), prior to completing the 1-back, participants were randomly assigned to either a Control condition, in which they received standard task instructions, or a Motivated condition, in which they were instructed that they would be allowed to leave the experimental session early if they performed sufficiently well on the 1-back.

#### 3.1.4 Self-reported motivation questions

Motivation was assessed before and after the 1-back task as well as on nine occasions throughout the 1-back using similar questions to those employed by Ralph et al. (2021). Specifically, participants were asked “How motivated [are/were] you to do well on the (1-back) task?”. Responses were provided using a 1 (not at all motivated) to 7 (very motivated) Likert scale. Motivation probes administered during the 1-back were pseudo-randomly positioned and set to occur once within each block of 52 trials.

#### 3.1.5 Motivation-related experiences

We also included three post-task questions concerning several experiences that might have been relevant to participants' feelings of motivation during the study. To assess their enjoyment of the 1-back, we asked, “How much did you enjoy completing the 1-back task?”. Response options for this question ranged from 1 (I did not enjoy it at all) to 7 (I enjoyed it very much). We also examined how interesting participants found the task using the question, “How interesting was it to complete the 1-back task?”, with response options ranging from 1 (not at all interesting) to 7 (very interesting). Finally, to further understand the impact of our motivation manipulation, participants in the Motivated condition were asked, “How motivated were you to do well on the 1-back task, given that you were allowed to leave early?”. Response options ranged from 1 (not at all motivated) to 7 (very motivated).

#### 3.1.6 Post-task multitasking questions

Since Study 2 was conducted online, we were concerned that participants might engage in activities outside the experimental context (i.e., beyond the 1-back task or optional video). To assess this, we administered a post-task question previously employed by Drody et al. (2022, 2024). Participants were asked, “Were you engaged in any tasks other than those related to the experiment?” and could select either “Yes” or “No.” They were then asked, “If so, did any of these activities involve the use of multimedia devices (e.g., smartphones, laptops, tablets, etc.)?”, with response options of “Yes,” “No,” or “Not applicable—I was completely on task the entire time.”

For the purposes of our data analysis and for consistency across Studies 1 and 2, we did not exclude any participants who indicated that they engaged in activities outside the experimental context. However, because it is possible that our pattern of effects could be impacted by individuals who engaged in alternate forms of multitasking or “purer” forms of media multitasking outside of the study context, for the sake of completion, we present the following analyses within Supplementary material: (1) when excluding participants who engaged in tasks outside of the experiment (i.e., they responded “Yes” to our first question), and (2) when only including participants who engaged in tasks outside of the experiment. Summaries of these findings are also described below in brief.

#### 3.1.7 Familiarity with the video

As in Ralph et al.'s (2021) study, we assessed participants' familiarity with the optional video using the question, “Have you seen the video used in this experiment before?”. Participants could respond with “Yes”, “No” or “Not applicable—I didn't watch the video”. Since only two participants in each condition reported having previously seen the video, responses to this question were not used for data filtering purposes and are not included in subsequent analyses.

### 3.2 Results

#### 3.2.1 Data pre-processing

To remain consistent with the data pre-processing methods employed in Ralph et al.'s (2021) study, participants with < 30% hits and >20% false alarms were removed prior to analysis of the data. Following data removal, our sample consisted of 284 participants (1 agender, 5 genderqueer, 45 men, 231 women, 2 no answer), with 154 participants in the Motivated condition and 130 participants in the Control condition. Based on the 279 responses to our age question, our final sample ranged from 17 to 44 years old (*M*_*age*_ = 20.00, *SD*_*age*_ = 3.40).

#### 3.2.2 Impact of motivation manipulation

**Figure 5:**
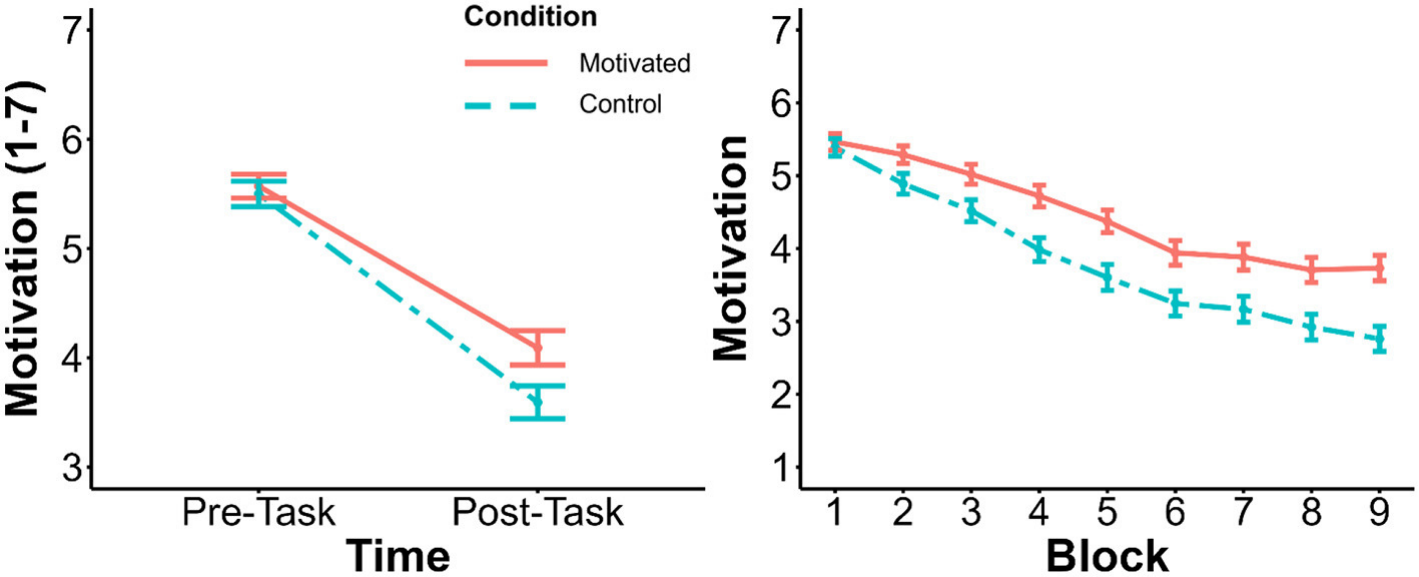
Graphs depicting mean levels of motivation before and after the 1-back **(left)** as well as over successive blocks of the 1-back **(right)** as a function of condition. Error bars represent +/– 1 standard error of the mean.

As in Study 1, we first examined pre- and post-task ratings of motivation using a mixed factorial ANOVA with Condition (Motivated or Control) as a between-participants factor and Time (Pre- or Post-Task) as a within-participants factor. No difference was found across conditions, *F*_(1,282)_ = 3.26, *p* = 0.072„ $\eta_{p}^{2}$ = 0.01. There was, however, a main effect of Time, *F*_(1,282)_ = 237.88, *p* < 0.001, $\eta_{p}^{2}$ = 0.46, with motivation levels decreasing from the start to the end of the task (Figure 5). The interaction between Condition and Time did not reach significance, *F*_(1,282)_ = 3.78, *p* = 0.053, $\eta_{p}^{2}$ = 0.01.

We next examined changes in motivation over the course of the 1-back task using a mixed factorial ANOVA with Condition as a between-participants factor and Block (corresponding to the nine probes in the 1-back) as a within-participants factor. Two participants with missing responses on one or more of the probes were removed from this analysis. Mauchly's test indicated that the assumption of sphericity was violated (*W* = 0.04, *p* < 0.001), therefore a Greenhouse-Geisser correction was applied (ε = 0.46). We observed a significant main effect of Condition, *F*_(1,270)_ = 10.60, *p* = 0.001, $\eta_{p}^{2}$ = 0.04, indicating that participants in the Motivated condition were significantly more motivated than those in the Control condition. We also observed a main effect of Block, *F*_(3.71,1001.49)_ = 138.13, *p* < 0.001, η^2^*p* = 0.34, with motivation decreasing over the course of the task. Additionally, there was an interaction between Condition and Block, *F*_(3.71,1001.49)_ = 3.64, *p* = 0.007, η^2^*p* = 0.01, such that participants in the Motivated condition began the task with similar levels of motivation as those in the Control condition, but they showed slower declines in motivation over time (Figure 5).

#### 3.2.3 Impact of motivation manipulation on media multitasking

**Figure 6:**
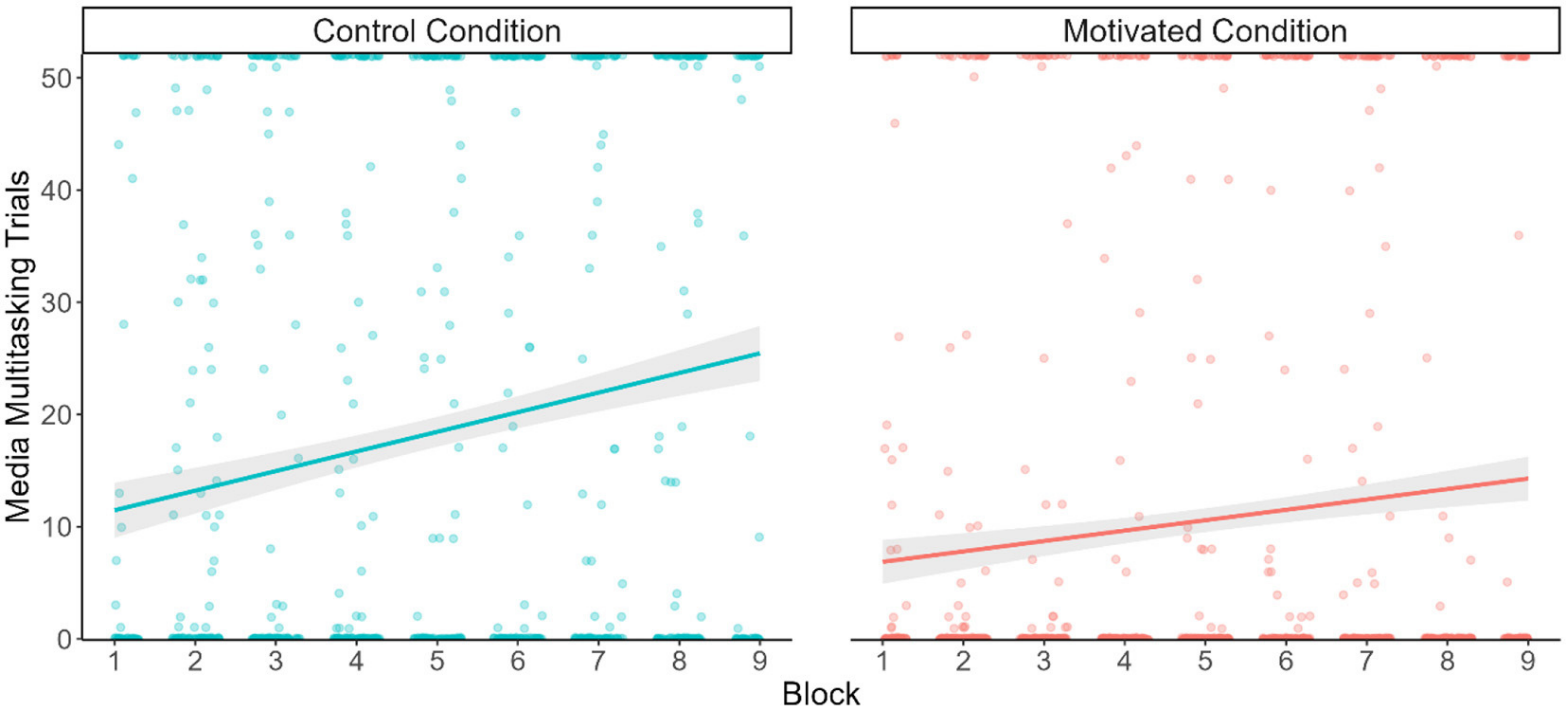
Scatterplots illustrating changes in media multitasking over successive blocks of the 1-back for those in the Control condition **(left)** and Motivation condition **(right)**. Data points are jittered to increase their visibility. Regression lines are fitted to the data and shading represents 95% confidence intervals.

As in Study 1, to examine changes in media multitasking over the course of the 1-back task, the trials of the task were divided into nine blocks of 52 trials. Within each block, we calculated the total number of trials during which participants engaged in media multitasking. Changes in media multitasking as a function of time and condition were assessed using a GLMM, with Condition (Control as the reference group) and Block (centered around zero) as fixed effects, and intercepts and slopes that were allowed to vary randomly by participant and Block. A Poisson distribution was specified for the outcome variable (media multitasking). Results of the GLMM are shown in Table 2. There was a significant effect of Condition such that those in the Motivated condition were less likely to media multitask than those in the Control condition. While there was a numeric decrease in motivation over successive blocks, the main effect of Block was not significant. There was, however, a significant interaction between Condition and Block, revealing that increases in media multitasking over time were more gradual for those in the Motivated condition compared to those in the Control condition (Figure 6). More information about participants' media multitasking behaviors can be found in the Supplementary material.

**Table 2:** GLMM results with block, condition and their interaction predicting media multitasking.

**Fixed effects**	**Estimate**	**SE**	***z***	***p***
Intercept	−0.20	0.41	−0.49	0.626
Condition (motivated group)	−2.34	0.57	−4.11	< 0.001
Block	0.01	0.05	0.21	0.832
Block^*^condition	−0.13	0.06	−2.09	0.04

N = 284. Coefficients for the effect of Condition are shown for the Motivated condition (Control is the reference group). P-values are based on Wald z-tests comparing estimates against zero.

#### 3.2.4 Changes in task performance over time

**Figure 7:**
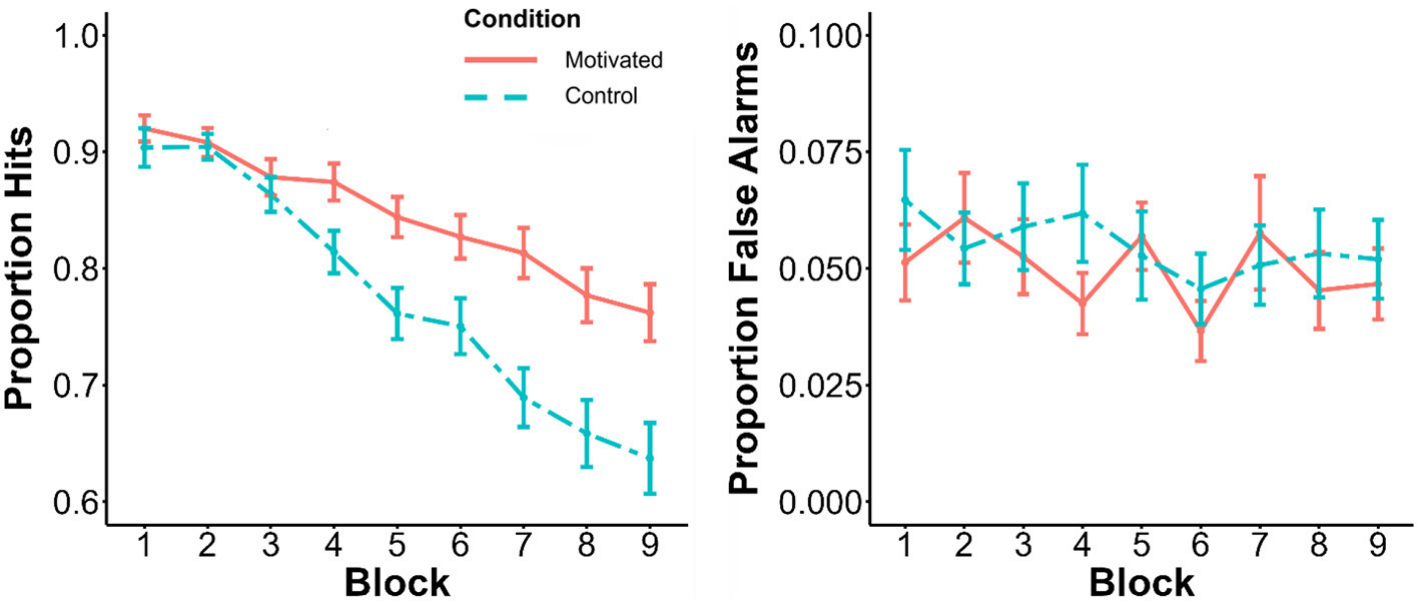
Line graphs depicting proportion hits **(left)** and proportion false alarms **(right)** over successive blocks of the 1-back for participants in the Motivated and Control condition. Error bars represent +/– 1 standard error of the mean.

As in Study 1, proportion hits and false alarms were calculated for each of the nine blocks of the 1-back (Figure 7) and changes in performance over time were evaluated using mixed factorial ANOVAs with either proportion hits or proportion false alarms as the dependent variable, Condition as a between-participants factor and Block as a within-participants factor. Mauchly's tests indicated that the assumption of sphericity was violated for the ANOVAs for both proportion hits (*W* = 0.55, *p* < 0.001) and proportion false alarms (*W* = 0.67, *p* < 0.001). Results are therefore presented using Greenhouse-Geisser estimates of sphericity (ε _*proportion hits*_ = 0.56, ε _*proportion false alarms*_ = 0.91).

Concerning proportion hits, there was a main effect of Condition, *F*_(1,282)_ = 10.72, *p* = 0.001, $\eta_{p}^{2}$ = 0.04, indicating that proportion hits were higher in the Motivated condition compared to the Control condition. Additionally, there was a main effect of Block, *F*_(4.43,1249.87)_ = 60.05, *p* < 0.001, $\eta_{p}^{2}$ = 0.18, such that proportion hits declined over time. A significant interaction between Condition and Block was also observed, *F*_(4.43,1249.87)_ = 5.77, *p* < 0.001, $\eta_{p}^{2}$ = 0.02, revealing that proportion hits decreased more gradually over time in the Motivated condition compared to the Control condition.

As in Study 1, proportion false alarms were at floor and no main effect of Condition, *F*_(1,282)_ = 0.71, *p* = 0.399, $\eta_{p}^{2}$ < 0.01, or Block, *F*_(7.26,2048.41)_ = 0.83, *p* = 0.565, $\eta_{p}^{2}$ < 0.01, was found. Moreover, there was no interaction between Condition and Block, *F*_(7.26,2048.41)_ = 0.61, *p* = 0.753, $\eta_{p}^{2}$ < 0.01. Analyses examining the potential impact of media multitasking on task performance can be found in the Supplemental material.

#### 3.2.5 Motivation-related experiences

Participants in the Motivated (*M* = 2.68, *SD* = 1.48) and Control (*M* = 2.64, *SD* = 1.53) conditions did not differ in terms of their enjoyment of the 1-back, *t*_(282)_ = 0.24, *p* = 0.809, *d* = 0.03 or how interesting they found the task, *t*_(282)_ = 0.34, *p* = 0.731, *d* = 0.04 (*M*_*Motivated*_ = 2.38, *SD*_*Motivated*_ = 1.52; *M*_*Control*_ = 2.45, *SD*_*Control*_ = 1.57). Overall, participants in the Motivated condition reported that they were motivated by the instructions informing them that they could leave the task early, scoring near the midpoint (*M* = 5.29, *SD* = 1.79) on our 7-point Likert scale, where 1 represented “not at all motivated” and 7 represented “very motivated”.

#### 3.2.6 Post-task multitasking questions

Of the 282 participants who provided complete responses to whether they had engaged in tasks other than those related to the experiment, 57 participants in the Control condition (~45%) and 56 participants in the Motivated condition (36%) responded “Yes”. Responses to this question did not differ across conditions, $X_{\left(1\right)}^{2}$ = 1.62, *p* = 0.204. Based on the 282 participants who provided complete responses to whether these activities involved the use of multimedia devices, 44 participants in the Control condition (34%) and 48 participants in the Motivated condition (31%) responded “Yes”. Once again, responses did not differ across conditions, $X_{\left(2\right)}^{2}$ = 4.94, *p* = 0.085.

When re-analyzing the data excluding participants who engaged in activities outside the experimental context (*N* = 169, 71 Control, 98 Motivated) and when re-analyzing the data for participants who reported engaging in other activities during the experiment (*N* = 113, 57 Control, 56 Motivated), results were similar. Patterns for these subgroups resembled the patterns observed in our full sample, although certain effects did not reach significance. Differences between conditions were particularly small in the subsample who reported engaging in tasks other than those related to the experiment. The results of these analyses are described in greater detail within the Supplementary material.

### 3.3 Discussion

Study 2 aimed to replicate the finding that increasing motivation slows temporal increases in media multitasking and examine how self-reported levels of motivation vary with changes in media multitasking over time. Despite participants in both conditions frequently reporting engaging in activities outside the experimental context, results largely resembled those of Study 1. That is, we observed more gradual increases in media multitasking as well as slower reductions in performance over time for participants in the Motivated condition relative to those in the Control condition. Regarding participants' experiences of motivation, while both conditions began the study with similar levels of motivation, those in the Motivated condition showed slower declines in motivation over time. Moreover, participants in the Motivated condition reported being specifically motivated by the possibility of leaving the experiment early, although their interest in and enjoyment of the 1-back did not differ from participants in the Control condition. These results largely align with the view that temporal changes in motivation are related to changes in media multitasking over time. Moreover, they suggest that minimizing decreases in motivation over time may be an effective strategy for mitigating temporal increases in media multitasking and accompanying performance declines.

It should be noted that conducting the same analyses after excluding participants who engaged in activities outside of the experiment, as well as after only including participants who engaged in activities outside the experimental context, produced a similar pattern of results. Although certain effects did not reach significance in these smaller subsets, interestingly, differences in media multitasking and motivation across conditions were smaller in the group of participants who reported engaging in activities outside the experimental context, suggesting that our manipulation of motivation was less effective in this group. While it is not entirely surprising that splitting our sample into smaller subsets led to smaller and sometimes non-significant effects, the discrepancy between results involving our full sample and our subsamples might suggest that the effect of manipulating motivation on temporal changes in media multitasking, performance, and self-reported motivation is quite subtle. We address this further in the General Discussion.

## 4 General discussion

A well-established finding in the vigilance literature is that attention and performance decline as time-on-task increases (Risko et al., 2012; Thomson et al., 2014; Yanko and Spalek, 2014; Krimsky et al., 2017; Brosowsky et al., 2023). In modern contexts, temporal declines in attention can manifest as increases in media multitasking (Wammes et al., 2019; Drody et al., 2024). Across two studies, we investigated whether increasing individuals' motivation to attend to a primary task could mitigate temporal increases in media multitasking and associated performance declines. Consistent with prior research (Wammes et al., 2019; Drody et al., 2024), results showed that media multitasking increased, and task performance decreased over time. Importantly, these changes were less pronounced among participants who received motivating instructions compared to those given standard instructions. Furthermore, Study 2, which additionally probed motivation throughout the 1-back task, revealed that self-reported motivation decreased with time-on-task. Once again, this decline was attenuated among participants who received motivating instructions.

Findings from these studies suggest that changes in media multitasking over time are closely tied to changes in motivation. This interpretation is congruous with prior research demonstrating that motivation decreases over the course of sustained attention tasks (Möckel et al., 2015; Melo et al., 2017; Brosowsky et al., 2023), often alongside decreases in attention to the task (Brosowsky et al., 2023) and task performance (Möckel et al., 2015; Melo et al., 2017; Brosowsky et al., 2023). Additionally, while previous studies have explored strategies to reduce sustained attention decrements by increasing motivation (Esterman et al., 2014, 2016; Hopstaken et al., 2015, 2016; Robison and Nguyen, 2023), this research is, to our knowledge, the first to demonstrate that increasing motivation can specifically mitigate temporal increases in media multitasking as well as corresponding performance declines.

Why might increasing motivation effectively reduce temporal increases in media multitasking? The media multitasking literature often describes this behavior as the outcome of a cost-benefit analysis, in which individuals weigh the value of focusing on their current task vs. alternative activities. For example, Drody et al. (2024) demonstrated that media multitasking becomes more likely as the opportunity costs of attending to one's current task rise over time (Kurzban et al., 2013). Interestingly, they also found that rising boredom accompanied increases in media multitasking over time, which is reminiscent of the temporal decrease in motivation observed in Study 2. From an opportunity costs perspective, changes in these states over time may encourage individuals to shift their focus away from their current task toward more appealing alternatives (Kurzban et al., 2013).

Similarly, Wiradhany et al. (2021) posit that media multitasking occurs when individuals transition from a state of exploitation, during which their focus is maintained on the task-at-hand, to one of exploration, characterized by a search for alternative activities. Many factors are theorized to drive this shift, including a desire to achieve an optimal level of arousal, situational affordances (e.g., multitasking simply because they have the opportunity to do so), and a perception that alternative activities are more rewarding than one's current task (Wiradhany et al., 2021). Supporting this framework, individuals have been shown to engage in media multitasking to satisfy various needs, including those surrounding emotion regulation, socializing, and information-seeking (Wang and Tchernev, 2012; Hwang et al., 2014; Kononova and Chiang, 2015). Applying this perspective to temporal changes in media multitasking, it is possible that these needs become increasingly salient over time, leading individuals to perceive alternative activities as more rewarding than their current task. Overall, it seems likely that temporal increases in media multitasking can stem from a growing imbalance between the costs and benefits one associates with their current tasks vs. other possible activities. Critically, however, increasing individuals' motivation to perform well on a task might encourage them to prioritize their current task over appealing alternatives, enabling them to sustain their attention on the task for longer.

Importantly, temporal increases in media multitasking represent a single example of how attention to a primary task and performance declines over time. Indeed, sustained attention decrements commonly occur in the absence of opportunities to media multitask (e.g., Davies and Parasuraman, 1982; Grier et al., 2003; Helton and Russell, 2011; Krimsky et al., 2017; Mackworth, 1948; Smit et al., 2004; Warm et al., 2008). For example, when other tasks are unavailable, sustained attention decrements may be associated with increases in off-task thought (Risko et al., 2012; Thomson et al., 2014; Yanko and Spalek, 2014; Brosowsky et al., 2023). Nevertheless, the implications of the present studies could extend beyond media multitasking to these broader forms of sustained attention. That is, the present findings suggest that attention to a primary task may not simply wane over time due to a gradual depletion of cognitive resources, as many have suggested (Grier et al., 2003; Caggiano and Parasuraman, 2004; Smit et al., 2004; Warm et al., 2008; Helton and Russell, 2011; Ross et al., 2014; Greenlee et al., 2024). Instead, or in addition, temporal changes in attention may be influenced by transient factors, such as the value individuals associate with current vs. alternative activities, which lead to a shift in attention away from one's current task toward off-task thought or other activities like media multitasking.

Some limitations are worth addressing with regard to the present work and its applications. The first limitation is that, although we discuss our findings in relation to theories of sustained attention decrements, the tasks employed in the present studies differed from those traditionally used in the vigilance literature. Traditional vigilance tasks typically require participants to detect and respond to infrequent events over extended periods of time which may last several hours (e.g., Mackworth, 1948; See et al., 1995; McBride et al., 2007; Pattyn et al., 2008). In contrast, our paradigm involved a working memory task that required participants to sustain their attention for ~20 min. Despite these differences, we observed decreases in attention and performance with time-on-task in both studies. This was unsurprising given that similar declines have been observed across traditional vigilance tasks (Mackworth, 1948; See et al., 1995; Grier et al., 2003; McBride et al., 2007; Pattyn et al., 2008), abbreviated vigilance tasks (e.g., Nuechterlein et al., 1983; Temple et al., 2000) and sustained attention tasks ranging from 5 to 30 min in duration (Thomson et al., 2014; Yanko and Spalek, 2014; Brosowsky et al., 2023). It is important to note though our goal was not to replicate the effects observed in traditional vigilance studies, but to instead consider the possibility that increases in media multitasking over time represent another example of sustained attention decrements and explore whether motivation would mitigate these increases and accompanying performance costs. In pursuing this goal, we found evidence that increasing motivation can help mitigate both temporal changes in media multitasking and decreases in performance over time.

A second limitation is that nearly half of our sample in Study 2 reported media multitasking with activities outside the experimental context. As a result, we additionally examined the data after excluding participants who reported engaging in these activities in order to obtain a more controlled measure of media multitasking akin to what might be observed in a laboratory setting. We also separately analyzed the data for those who reported engaging in other activities to understand the impact of real-world media multitasking on our measures. Overall, patterns of media multitasking, self-reported motivation, and performance over time were similar across our full sample and the two subsamples; however, some effects no longer reached statistical significance in our subsamples. We interpret the differences between results involving the full and reduced samples as suggesting that increasing motivation at the start of a task effectively boosts motivation, decreases media multitasking, and improves performance throughout the task. However, the extent to which manipulating motivation influences changes in these variables over time may be subtle. Future research could further investigate the robustness of these findings.

It should also be noted that our studies explored media multitasking under specific circumstances in which participants were asked to complete a monotonous (1-back) task, presumably with their only options being to attend to the task or watch the video we provided. It is therefore possible that the observed changes in motivation and media multitasking over time were somewhat dependent on the nature of our paradigm. While previous studies, like ours, have reported steady increases in media multitasking over time in both laboratory (Drody et al., 2024) and real-world settings (a class lecture; Wammes et al., 2019), others have observed that this behavior fluctuates over time (Rosen et al., 2013; Ragan et al., 2014), suggesting that media multitasking behavior may be somewhat context-dependent. Interestingly however, despite conducting our two studies in different settings, Study 1 in the laboratory and Study 2 in a less constrained online environment where participants frequently reported engaging in activities outside the experimental context, findings were remarkably consistent across studies, pointing to the robustness of our overall patterns.

Another limitation concerns our manipulation of motivation. Previous studies have effectively motivated participants by informing them that the duration of their experimental session depended on their task performance (Esterman et al., 2014, 2016; Hopstaken et al., 2015, 2016; Seli et al., 2019; Ralph et al., 2021); however, similar manipulations may not be feasible in certain contexts. For example, settings like classrooms and workplaces often require individuals to remain present for fixed periods, limiting the applicability of manipulations of this kind. Alternative methods of increasing motivation in the laboratory have included incentivizing participants using point systems (Robison and Nguyen, 2023) or monetary rewards (Esterman et al., 2016), both of which have been shown to increase task performance compared to when no motivation manipulation is employed. In academic settings, researchers have found that gamification and the use of leaderboards can increase the amount of time students spend focused on assigned tasks (Landers and Landers, 2014). To deepen our understanding of the relation between motivation and sustained attention, future research could explore how different motivation manipulations impact temporal changes in media multitasking, and attention more broadly, across a variety of settings.

Sustained attention decrements, characterized by gradual reductions in attention and task performance over time, can manifest as increases in media multitasking at the expense of decreased task performance over time. Across two studies, we investigated whether increasing motivation could mitigate increases in media multitasking over the course of an experimental task. Media multitasking was found to increase with time-on-task while performance decreased; however, these trends were attenuated in participants who received motivating vs. standard task instructions. Additionally, decreases in self-reported motivation were found to accompany increases in media multitasking and decreases in performance over time. These decreases in motivation occurred more gradually for those who received the motivating instructions compared to those who received the standard instructions. In all, findings from the present studies suggest that temporal changes in media multitasking may be linked to changes in motivational factors, and that increasing motivation can improve individuals' ability to sustain their attention, presumably by increasing the value they assign to attending to current vs. alternative activities. These findings also align with accounts that attribute sustained attention decrements to varying cost-benefit analyses rather than a gradual depletion of resources over time.

## Data Availability

The datasets presented in this study can be found in online repositories. The names of the repository/repositories and accession number(s) can be found below: https://osf.io/xapbt/.

## Footnotes

^1^Ralph et al. (2021) originally reported having 82 participants in the Control condition and 84 participants in the Motivated condition. It appears that there was a typo in the original article such that the sample sizes were switched between conditions.
