# Supplementary material for: Inattention over time-on-task: the role of motivation in mitigating temporal increases in media multitasking
Source: Front Cognit. 2025 May 6;4:1547295. doi: 10.3389/fcogn.2025.1547295 (PMC13281209; doi:10.3389/fcogn.2025.1547295)
Supplement: Supplementary file 1 [file Data_Sheet_1.pdf]

Study 2 Results Excluding Participants Who Engaged in Activities Outside the Experimental Context

Data Preprocessing

After applying the data preprocessing methods employed in Ralph et al. (2021), which involved removing participants with less than 30% hits and greater than 20% false alarms, we filtered the data so that only participants who responded "No" to our question, "Were you engaged in any tasks other than those related to the experiment?" remained. The final sample consisted of 169 participants (137 women, 27 men, 2 genderqueer, 1 agender and 2 who preferred not to answer), with 71 participants in the Control condition and 98 participants in the Motivated condition. Based on the 168 complete responses to our age question, the age of our sample ranged from 17 to 44 years old (M_{age} = 20.22, *SD* = 3.78).

Motivation

We first examined pre- and post-task ratings of motivation using a mixed factorial ANOVA with Condition (Motivated or Control) as a between-participants factor and Time (Pre- or Post-Task) as a within-participants factor. The main effect of Condition neared, but did not reach significance, $F(1, 167) = 3.78$, $p = .053$, , $\eta^2_p = 0.02$; however, there was a main effect of Time, $F(1, 167) = 128.81$, $p < .001$, $\eta^2_p = 0.44$, with motivation levels decreasing from the start to the end of the task (Figure 1). There was no significant interaction between Condition and Time, $F(1, 167) = 1.54$, $p = .217$, $\eta^2_p = 0.01$.

We next examined changes in motivation over the course of the 1-back task using a mixed factorial ANOVA with Condition as a between-participants factor and Block (the nine probes in the 1-back) as a within-participants factor. Two participants with missing responses on one or more of the probes were removed from this analysis. Mauchly's test indicated that the assumption of

sphericity was violated (*W* = 0.05, $p < .001$), therefore, results are presented after applying a Greenhouse-Geisser correction ($\varepsilon = 0.50$). We observed a significant main effect of Condition, $F(1, 161) = 8.74$, $p = .004$, $\eta^2_p = 0.05$, indicating that participants in the Motivated condition were significantly more motivated than those in the Interest condition. We also observed a main effect of Block, $F(4.04, 649.92) = 74.30$, $p < .001$, $\eta^2_p = 0.32$, with motivation decreasing over the course of the task. The interaction between Condition and Block was not significant, $F(4.04, 649.92) = 1.90$, $p = .108$, $\eta^2_p = 0.01$ (Figure S1).

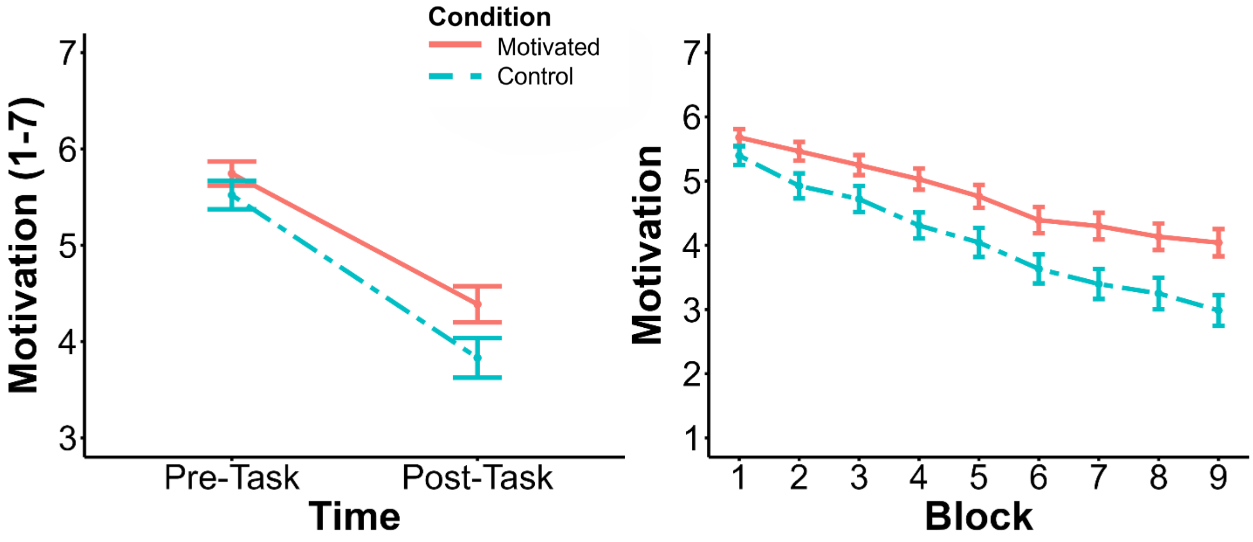

Figure S1. Graphs depicting mean levels of motivation before and after the 1-back (left) as well as over successive blocks of the 1-back (right) as a function of condition. Error bars represent +/- 1 Standard Error of the Mean.

Motivation-related Experiences

Participants in the Motivated ($M = 2.95$, $SD = 1.45$) and Control ($M = 2.90$, $SD = 1.57$) conditions did not differ in terms of their enjoyment of the 1-back, $t(167) = 0.20$, $p = .839$, $d = .03$ or how interesting they found the task, $t(167) = 0.02$, $p = .984$, $d = .00$ ($M_{Motivated} = 2.64$, $SD_{Motivated} = 1.53$; $M_{Control} = 2.65$, $SD_{Control} = 1.76$). Overall, participants in the Motivated condition reported that they were motivated by the instructions informing them that they could leave the task early, scoring

near the midpoint on our 7-point Likert scale (M = 5.54, SD = 1.57), where 1 represented "not at all motivated" and 7 represented "very motivated".

Impact of Motivation Manipulation on Media Multitasking

The trials of the 1-back were divided into nine blocks of 52 trials, corresponding to the number of motivation probes included in the task. Within each block, we calculated the total number of trials participants spent with the video on. Changes in media multitasking as a function of time and condition were assessed using a GLMM, with Condition (Control as the reference group) and Block (centered around zero) as fixed effects, and intercepts and slopes that were allowed to vary randomly by participant and Block. A Poisson distribution was specified for the outcome variable (media multitasking). Results of the GLMM are shown in Table S1 and changes in media multitasking over time are plotted for both conditions in Figure S2. There was a significant effect of Condition such that those in the Motivated condition were less likely to media multitask than those in the Control condition. There was no significant effect of Block, and there was no interaction between Condition and Block.

Table S1.

GLMM results with Block, Condition and their interaction predicting media multitasking

Fixed Effects	Estimate	SE	z	p
Intercept	-0.41	0.62	-0.66	.508
Condition (Motivated Group)	-3.03	0.83	-3.65	< .001
Block	-0.03	0.08	-0.34	.732
Block*Condition	-0.17	0.09	-1.81	.071

Note. N = 169. Coefficients for the effect of Condition are shown for the Motivated condition (Control is the reference condition). P-values are based on Wald-z tests comparing estimates against zero.

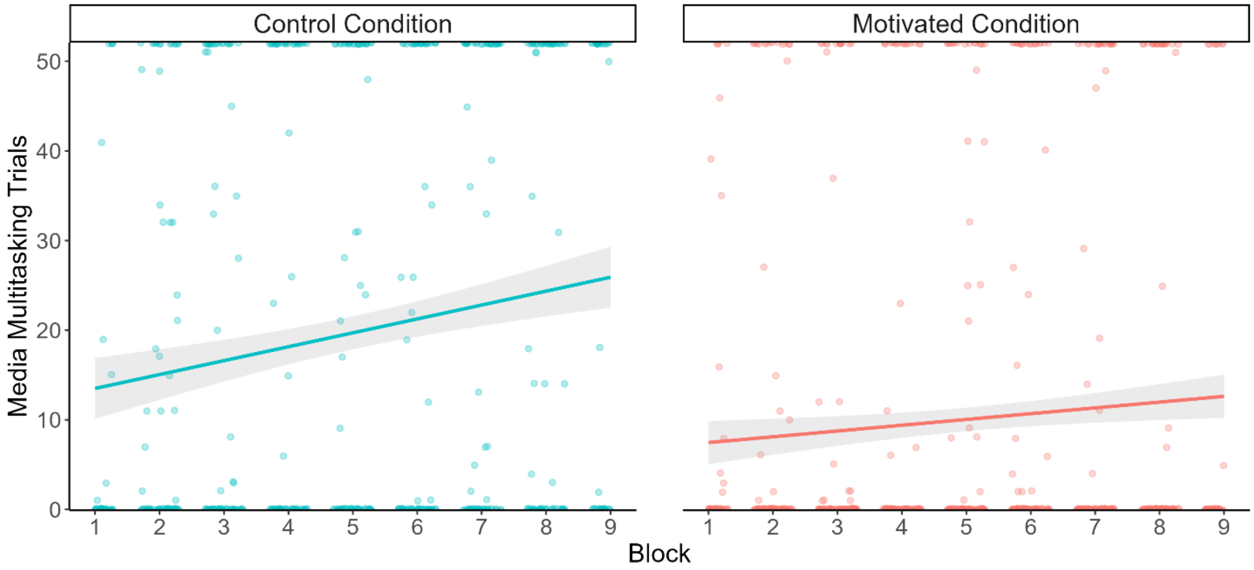

Figure S2. Scatterplots depicting changes in media multitasking over successive blocks of the 1-back for participants in the Control condition (left) and Motivation condition (right). Data points are jittered to increase their visibility. Regression lines are fitted to the data and shading represents 95% confidence intervals.

Changes in Performance Over Time

Proportion hits and false alarms were calculated for each of the nine blocks of the 1-back (Figure S3) and changes in performance over time were evaluated using mixed factorial ANOVAs with either proportion hits or proportion false alarms as the dependent variable, Condition as a between-participants factor and Block as a within-participants factor. Mauchly's tests indicated that the assumption of sphericity was violated for the ANOVAs involving both proportion hits (W = 0.18, p < .001) and proportion false alarms (W = 0.39, p < .001). Results are therefore presented using Greenhouse-Geisser estimates of sphericity ($\varepsilon_{proportion\ hits}$ = 0.60, $\varepsilon_{proportion\ false\ alarms}$ = 0.78).

Concerning proportion hits, there was a main effect of Condition, F(1, 167) = 5.11, p = .025, η^2_p = .03, indicating that proportion hits were higher in the Motivated condition compared to the Control condition. Additionally, there was a main effect of Block, F(4.80, 802.14) = 24.71, p < .001, η^2_p = .13, such that proportion hits declined over time. There was no significant interaction between

Condition and Block, $F(4.80, 802.14) = 1.98$, $p = .082$, $\eta^2_p = .01$. As in Study 1, proportion false alarms were at floor and no main effect of Condition, $F(1, 167) = 0.06$, $p = .806$, $\eta^2_p = .00$, or Block, $F(6.23, 1039.87) = 0.52$, $p = .796$, $\eta^2_p = .00$, was found. Moreover, there was no interaction between Condition and Block, $F(6.23, 1039.87) = 0.75$, $p = .615$, $\eta^2_p = .00$.

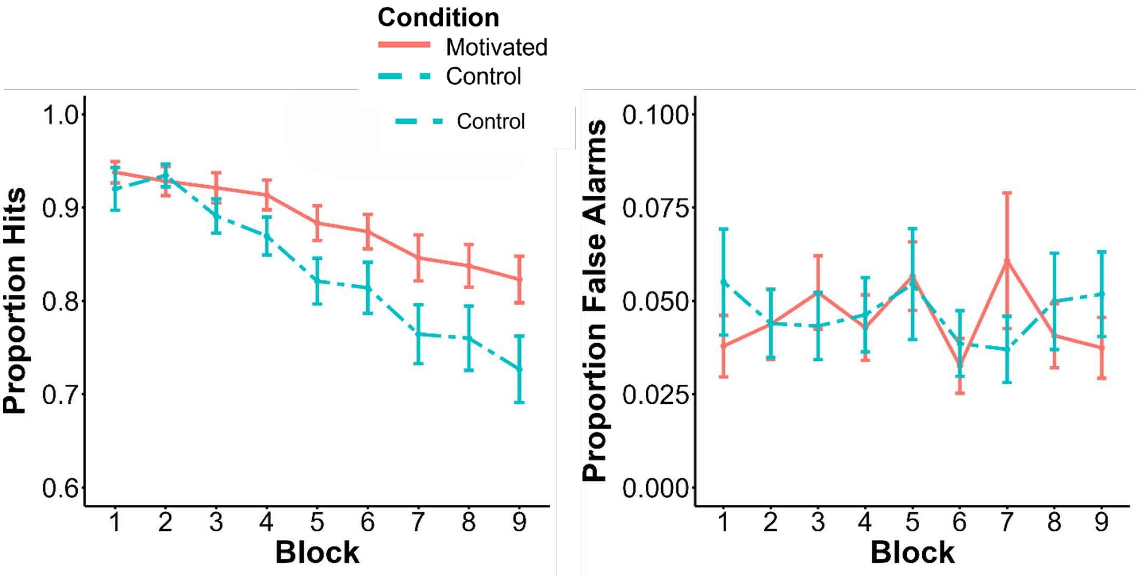

Figure S3. Line graphs depicting proportion hits (left) and proportion false alarms (right) over successive blocks of the 1-back for participants in the Motivated and Control condition. Error bars represent +/- 1 Standard Error of the Mean.

Study 2 Results Including Only Participants Who Engaged in Activities Outside the Experimental Context

Data Preprocessing

After applying the data preprocessing methods employed in Ralph et al. (2021), which involved removing participants with less than 30% hits and greater than 20% false alarms, we filtered our data so that only participants who responded "Yes" to our question, "Were you engaged in any tasks other than those related to the experiment?" remained. The final sample consisted of 113 participants (93 women, 17 men, 3 genderqueer), with 57 participants in the Control condition and 56 participants in the Motivated condition. Based on the 105 complete responses to our age question, the age of our sample ranged from 17 to 33 years old (M_{age} = 19.67, *SD* = 2.73).

Motivation

We first examined pre- and post-task ratings of motivation using a mixed factorial ANOVA with Condition (Motivated or Control) as a between-participants factor and Time (Pre- or Post-Task) as a within-participants factor. There was no main effect of condition, $F(1, 1111) = 0.02$, $p = .894$, , $\eta^2_p = 0.00$; however, there was a main effect of Time, $F(1, 111) = 101.79$, $p < .001$, $\eta^2_p = 0.48$, with motivation levels decreasing from the start to the end of the task (Figure S4). There was no interaction between Condition and Time, $F(1, 111) = 1.18$, $p = .280$, $\eta^2_p = 0.01$.

We next examined changes in motivation over the course of the 1-back task using a mixed factorial ANOVA with Condition as a between-participants factor and Block (corresponding to the nine probes in the 1-back) as a within-participants factor. Five participants with missing responses on one or more of the probes were removed from this analysis. As Mauchly's test indicated that the assumption of sphericity was violated ($W = 0.03$, $p < .001$), results are presented after applying a

Greenhouse-Geisser correction ($\varepsilon = 0.42$). There was no main effect of Condition, $F(1, 105) = 0.83$, $p = .363$, $\eta^2_p = 0.01$, although there was a main effect of Block, $F(3.34, 350.64) = 61.97$, $p < .001$, $\eta^2_p = 0.37$, with motivation decreasing over the course of the task. The interaction between Condition and Block was not significant, $F(3.34, 350.64) = 1.27$, $p = .283$, $\eta^2_p = 0.01$ (Figure S4).

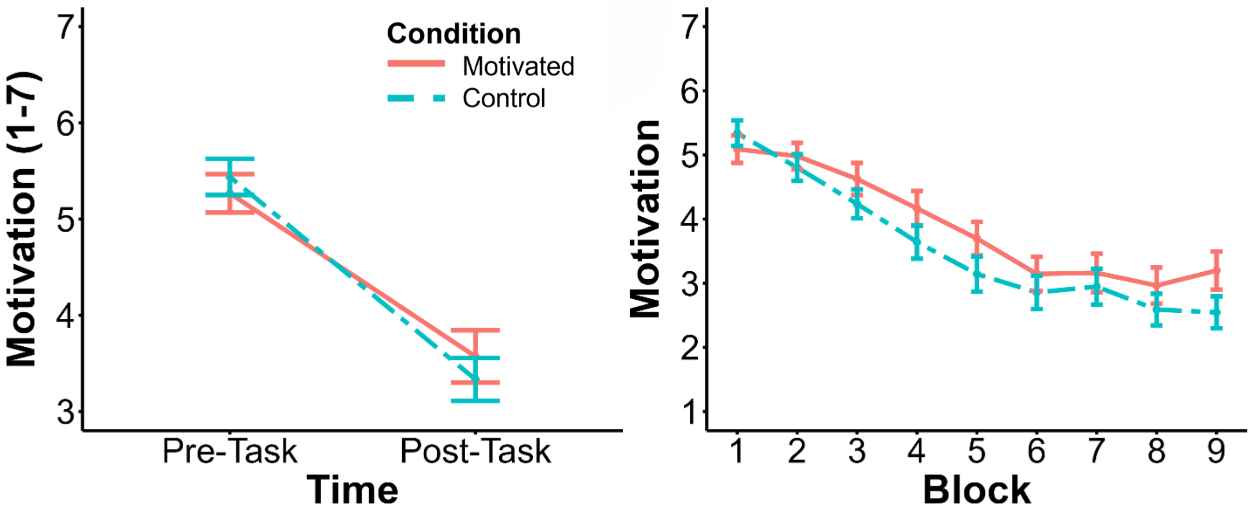

Figure S4. Graphs depicting mean levels of motivation before and after the 1-back (left) as well as over successive blocks of the 1-back (right) as a function of condition. Error bars represent +/- 1 Standard Error of the Mean.

Motivation-related Experiences

Participants in the Motivated ($M = 2.21$, $SD = 1.42$) and Control ($M = 2.32$, $SD = 1.45$) conditions did not differ in terms of their enjoyment of the 1-back, $t(111) = 0.37$, $p = .708$, $d = .07$ or how interesting they found the task, $t(111) = 0.98$, $p = .328$, $d = .18$ ($M_{Motivated} = 1.93$, $SD_{Motivated} = 1.39$; $M_{Control} = 2.18$, $SD_{Control} = 1.28$). Overall, participants in the Motivated condition reported that they were motivated by the instructions informing them that they could leave the task early, scoring near the midpoint on our 7-point Likert scale ($M = 4.88$, $SD = 2.07$), where 1 represented "not at all motivated" and 7 represented "very motivated".

Impact of Motivation Manipulation on Media Multitasking

The trials of the 1-back were divided into nine blocks of 52 trials to correspond to the number of probes included in the 1-back. Within each block, we calculated the total number of trials during which participants engaged in media multitasking. Changes in media multitasking as a function of time and condition were assessed using a GLMM, with Condition (Control as the reference group) and Block (centered around zero) as fixed effects, and intercepts and slopes that were allowed to vary randomly by participant and Block. A Poisson distribution was specified for the outcome variable (media multitasking). Results of the GLMM are shown in Table S2 and changes in media multitasking over time are plotted for both conditions in Figure S5. There was a significant effect of Condition such that those in the Motivated condition were less likely to media multitask than those in the Control condition. There was no significant effect of Block nor was there a significant interaction between Condition and Block.

Table S2.

GLMM results with Block, Condition and their interaction predicting media multitasking

Fixed Effects	Estimate	SE	*z*	*p*
Intercept	0.22	0.52	0.43	.668
Condition (Motivated Group)	-1.62	0.75	-2.16	.031
Block	0.07	0.06	1.13	.259
Block*Condition	-0.08	0.08	-0.94	.347

Note. *N* = 113. Coefficients for the effect of Condition are shown for the Motivated condition (Control is the reference condition). *P*-values are based on Wald *z*-tests comparing estimates against zero.

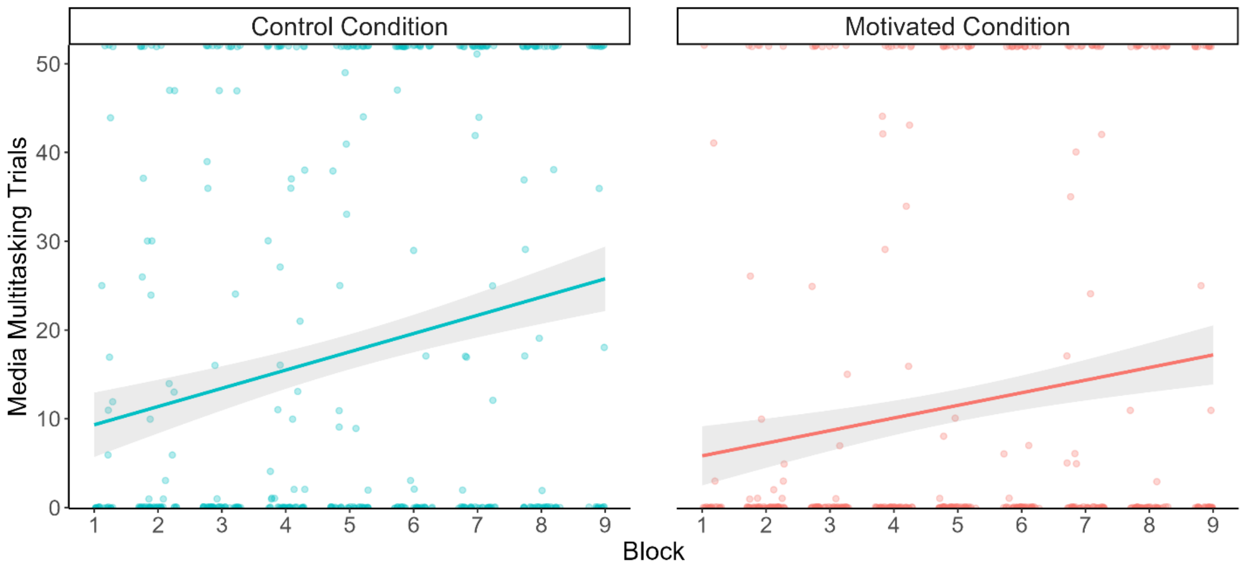

Figure S5. Scatterplots depicting changes in media multitasking over successive blocks of the 1-back for participants in the Control condition (left) and Motivation condition (right). Data points are jittered to increase their visibility. Regression lines are fitted to the data and shading represents 95% confidence intervals.

Changes in Performance Over Time

Proportion hits and false alarms were calculated for each of the nine blocks of the 1-back (Figure S6) and changes in performance over time were evaluated using mixed factorial ANOVAs with either proportion hits or proportion false alarms as the dependent variable, Condition as a between-participants factor and Block as a within-participants factor. Mauchly's tests indicated that the assumption of sphericity was violated for the ANOVAs involving both proportion hits (W = 0.10, $p < .001$) and proportion false alarms (W = 0.50, $p < .001$). Results are therefore presented using Greenhouse-Geisser estimates of sphericity ($\varepsilon_{proportion\ hits}$ = 0.54, $\varepsilon_{proportion\ false\ alarms}$ = 0.87).

Concerning proportion hits, there was no main effect of Condition, $F(1, 111) = 3.02$, p = .085, η^2_p = .03. We did observe a main effect of Block, $F(4.31, 478.13) = 33.55$, $p < .001$, η^2_p = .23, such that proportion hits declined over time. There was a significant interaction between Condition and Block, $F(4.31, 478.13) = 2.66$, $p = .029$, η^2_p = .02. Proportion false alarms were at floor and no

main effect of Condition, $F(1, 111) = 0.23$, $p = .631$, $\eta^2_p = .00$, or Block, $F(6.95, 771.12) = 1.18$, $p = .314$, $\eta^2_p = .01$, was found. Moreover, there was no interaction between Condition and Block, $F(6.95, 771.12) = 0.96$, $p = .458$, $\eta^2_p = .01$.

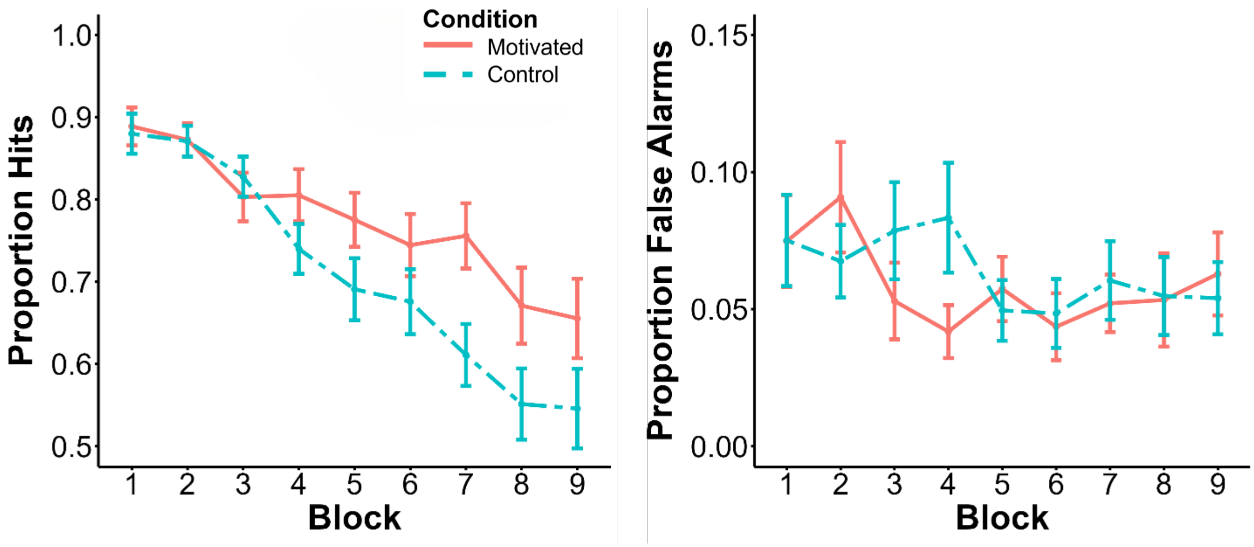

Figure S6. Line graphs depicting proportion hits (left) and proportion false alarms (right) over successive blocks of the 1-back for participants in the Motivated and Control condition. Error bars represent +/- 1 Standard Error of the Mean.

Additional Analyses for Study 1 (Full Sample)

Video Switching Patterns

The number of times participants switched the video from an "off" to an "on" state ranged from 0 to 7. Approximately 34% of participants switched the video on only once, while approximately 25% switched it on more than once. There was no difference in rates of switching between the Control (M = 1.08, SD = 1.19) and Motivated (M = 1.08, SD = 1.42) conditions.

Correlations Involving Motivation, Media Multitasking and Performance

Table S3.

Correlations involving motivation ratings, media multitasking and 1-back performance

	Pre-Task Motivation	Post-Task Motivation	Media Multitasking	Proportion Hits	Proportion False Alarms
Pre-Task Motivation		.64***	-.25*	.18	.04
Post-Task Motivation	.61***		-.11	.26*	-.08
Media Multitasking	-.16	-.04		-.40***	.25*
Proportion Hits	.28*	.31**	-.29**		-.40***
Proportion False Alarms	-.05	-.05	-.01	-.22	

Note. Relations for the Control condition are shown above the diagonal, and relations for the Motivated condition are presented below the diagonal. Due to the skewed nature of the media multitasking data, Spearman correlations were conducted for relations involving media multitasking. All other coefficients are Pearson correlations. **p* < .05, ***p* < .01, ****p* <.001.

The Impact of Media Multitasking on Performance

To examine whether media multitasking impacted performance, we conducted two mixed factorial ANOVAs. Trial Type, which differentiated trials with the video on versus off, was included as a within-participants factor and Condition (Motivated or Control) was included as a between-

participants factor. The dependent variable for each ANOVA was either proportion hits or proportion false alarms. Seventy-nine participants were omitted from this analysis, as they did not have both video on and video off trials for comparison. For proportion hits, there was no main effect of Condition, $F(1, 76) = 3.31$, $p = .073$, $\eta_p^2 = .04$, although there was a main effect of Trial Type, $F(1, 76) = 19.09$, $p < .001$, $\eta_p^2 = .20$, such that proportion hits were higher when the video was turned off than when it was turned on. There was no interaction between Condition and Trial Type, $F(1, 76) = 0.14$, $p = .714$, $\eta_p^2 = .00$. Regarding proportion false alarms, there was no main effect of Condition, $F(1, 76) = 2.82$, $p = .097$, $\eta_p^2 = .04$, no main effect of Trial Type, $F(1, 76) = 0.93$, $p = .338$, $\eta_p^2 = .01$, and no interaction between Condition and Trial Type, $F(1, 76) = 1.25$, $p = .267$, $\eta_p^2 = .02$. Table S4 presents descriptive statistics of performance as a function of Trial Type and Condition.

Table S4.

Descriptive Statistics of Performance as a Function of Condition and Trial Type

Condition	Trial Type	Proportion Hits<br>*M* (*SD*)	Proportion False Alarms<br>*M* (*SD*)
Control	Video Off<br>(n = 67)	0.89 (0.15)	0.02 (0.06)
	Video On<br>(n = 49)	0.77 (0.23)	0.01 (0.03)
Motivated	Video Off<br>(*n* = 77)	0.94 (0.08)	0.01 (0.02)
	Video On<br>(*n* = 42)	0.85 (0.19)	0.01 (0.01)

Additional Analyses for Study 2 (Full Sample)

Video Switching Patterns

The number of times participants switched the video from an "off" to an "on" state ranged from 0 to 7. Approximately 26% of participants switched the video on only once, and 13% of participants switched the video on multiple times. Switching was greater in the Control ($M = 0.79$, $SD = 1.22$) condition compared to the Motivated condition ($M = 0.47$, $SD = 0.76$), $t(282) = 2.74$, $p = .007$, $d = .33$.

Correlations Involving Motivation, Media Multitasking and Performance

Table S5.

Correlations involving motivation ratings, media multitasking and 1-back performance

	Pre-Task Motivation	Post-Task Motivation	Media Multitasking	Proportion Hits	Proportion False Alarms
Pre-Task Motivation		$.33^{***}$	.12	-.02	-.08
Post-Task Motivation	$.39^{***}$		-.02	$.40^{***}$	$-.24^{**}$
Media Multitasking	-.06	$-.21^{*}$		$-.22^{*}$	-.01
Proportion Hits	$.28^{***}$	$.54^{***}$	$-.17^{*}$		$-.29^{***}$
Proportion False Alarms	-.09	$-.19^{*}$	.09	$-.53^{***}$	

Note. Relations for the Control condition are shown above the diagonal, and relations for the Motivated condition are presented below the diagonal. Due to the skewed nature of the media multitasking data, Spearman correlations were conducted for relations involving media multitasking. All other coefficients are Pearson correlations. $^{*}p < .05$, $^{**}p < .01$, $^{***}p < .001$.

The Impact of Media Multitasking on Performance

To examine whether media multitasking impacted performance, we conducted two mixed factorial ANOVAs with Trial Type (video on versus off) as a within-participants factor and Condition (Motivated or Control) as a between-participants factor. The dependent variable for each

ANOVA was either proportion hits or proportion false alarms. One hundred and fifty-six participants were omitted from this analysis because they did not have both video on and video off trials for comparison. Concerning proportion hits, there was no main effect of Condition, $F(1, 126) = 1.29$, $p = .259$, $\eta_p^2 = .01$, although there was a main effect of Trial Type, $F(1, 126) = 38.73$, $p < .001$, $\eta_p^2 = .24$, with proportion hits being higher when the video was turned off rather than on. There was no interaction between Condition and Trial Type, $F(1, 126) = 0.40$, $p = .530$, $\eta_p^2 = .00$. Regarding proportion false alarms, there was no main effect of Condition, $F(1, 126) = 0.88$, $p = .351$, $\eta_p^2 = .01$, no main effect of Trial Type, $F(1, 126) = 0.28$, $p = .595$, $\eta_p^2 = .00$, and no interaction between Condition and Trial Type, $F(1, 126) = 0.00$, $p = .972$, $\eta_p^2 = .00$. Table S5 shows descriptive statistics of performance as a function of Trial Type and Condition.

Table S5.

Descriptive Statistics of Performance as a Function of Condition and Trial Type

Condition	Trial Type	Proportion Hits<br>*M* (*SD*)	Proportion False Alarms<br>*M* (*SD*)
Control	Video Off<br>(n = 143)	0.86 (0.17)	0.01 (0.01)
	Video On<br>(n = 67)	0.75 (0.31)	0.01 (0.01)
Motivated	Video Off<br>(*n* = 119)	0.84 (0.16)	0.01 (0.01)
	Video On<br>(*n* = 83)	0.68 (0.27)	0.01 (0.01)

References

Ralph, B. C. W., Smith, A. C., Seli, P., and Smilek, D. (2021). The relation between task-unrelated media multitasking and task-related motivation. *Psychol Res* 85, 408–422. doi: 10.1007/s00426-019-01246-7
